# Antiproliferative and Pro-Apoptotic Effect of Novel Nitro-Substituted Hydroxynaphthanilides on Human Cancer Cell Lines

**DOI:** 10.3390/ijms17081219

**Published:** 2016-07-28

**Authors:** Tereza Kauerova, Jiri Kos, Tomas Gonec, Josef Jampilek, Peter Kollar

**Affiliations:** 1 Department of Human Pharmacology and Toxicology, Faculty of Pharmacy, University of Veterinary and Pharmaceutical Sciences Brno, Palackeho 1946/1, 612 42 Brno, Czech Republic; tereza.kauerova@gmail.com; 2 Department of Chemical Drugs, Faculty of Pharmacy, University of Veterinary and Pharmaceutical Sciences Brno, Palackeho 1946/1, 612 42 Brno, Czech Republic; kosj@vfu.cz (J.K.); t.gonec@seznam.cz (T.G.); 3 Department of Pharmaceutical Chemistry, Faculty of Pharmacy, Comenius University, Odbojarov 10, 832 32 Bratislava, Slovakia; josef.jampilek@gmail.com

**Keywords:** hydroxynaphthanilides, salicylanilides, cell proliferation, apoptosis, anticancer effect

## Abstract

Ring-substituted hydroxynaphthanilides are considered as cyclic analogues of salicylanilides, compounds possessing a wide range of pharmacological activities, including promising anticancer properties. The aim of this study was to evaluate the potential anticancer effect of novel nitro-substituted hydroxynaphthanilides with a special focus on structure-activity relationships. The antiproliferative effect was assessed by Water Soluble Tetrazolium Salts-1 (WST-1) assay, and cytotoxicity was evaluated via dye exclusion test. Flow cytometry was used for cell cycle analysis and detection of apoptosis using Annexin V-FITC/PI assay. Protein expression was estimated by Western blotting. Our data indicate that the potential to cause the antiproliferative effect increases with the shift of the nitro substituent from the *ortho*- to the *para*-position. The most potent compounds, 3-hydroxy-*N*-(3-nitrophenyl)naphthalene-2-carboxamide (**2**), and 2-hydroxy-*N*-(4-nitrophenyl)-naphthalene-1-carboxamide (**6**) showed antiproliferative activity against THP-1 and MCF-7 cancer cells without affecting the proliferation of 3T3-L1 non-tumour cells. Compounds **2** and **6** induced the accumulation of THP-1 and MCF-7 cells in G1 phase associated with the downregulation of cyclin E1 protein levels, while the levels of cyclin B1 were not affected. Moreover, compound **2** was found to exert the pro-apoptotic effect on the THP-1 cells. These results suggest that hydroxynaphthanilides might represent a potential model structure for the development of novel anticancer agents.

## 1. Introduction

Salicylanilide derivatives (*N*-substituted hydroxybenzamides) are known as multitarget agents that possess a wide spectrum of pharmacological activities. These compounds are largely investigated for their promising antibacterial and antimycobacterial effects [1,2,3,4,5]. Some salicylanilides, such as niclosamide or closantel, belong to the class of broad-spectrum anthelmintic agents [6]. Recently, using high-throughput screening, several studies uncovered an antitumor activity of niclosamide, thereby becoming widely studied as a potential anticancer agent [7]. It was proved to effectively induce growth inhibition in a broad spectrum of tumour cell lines together with a minimal toxicity on non-tumour cells [8,9]. On the molecular level, niclosamide inhibited multiple key oncogenic signalling pathways (e.g., Wnt/β-catenin, mTORC1, and NF-κB) [9,10,11,12]. In general, salicylanilide derivatives are presumed to share the structure similarity with the pharmacophore of 4-arylaminoquinazoline derivatives (e.g., gefitinib and erlotinib) that belong to the class of small molecule inhibitors of the protein kinase epidermal growth factor receptor (EGFR PTK) [13,14,15]. This fact led to the intensive research of salicylanilides anticancer properties, as their structure became an attractive model for the design of potent antitumor agents. Several studies were published, in which a series of newly-prepared salicylanilides showed antiproliferative activity against a spectrum of human cancer cell lines, such as promyelocytic leukaemia cells HL-60, chronic myelogenous leukaemia cells K562, human epithelial carcinoma cells A431, or breast carcinoma cells MCF-7. In addition, some salicylanilides have been recently reported to elicit cell cycle arrest or to induce apoptosis in human cancer cell lines [13,16,17,18].

Recently, several series of various ring-substituted hydroxynaphthanilides were designed and prepared as ring analogues of salicylanilides. Based on the principle of bioisosterism with quinoline-like compounds, the aromatic ring in the salicylanilide pharmacophore was extended by another to obtain the naphthalene scaffold in the structure [3,5]. Compounds containing a quinoline moiety exhibit various pharmacological effects, including anticancer activity [19], hence the hydroxynaphthanilides may possess promising pharmacological properties due to the connection of these two pharmacophores.

The biological activity of salicylanilide pharmacophore could be modified by introducing appropriate substituents in the structure. In addition to a substitution pattern on the salicylic scaffold, SAR studies were focused also on substituents located on the aromatic ring of the anilide part in the structure. It was proved that the biological effects of salicylanilide derivatives are related to both the nature and the position of substituents. The electron parameters of anilide substituents could modify the conformational equilibrium between the closed-ring and open-ring forms of the structure and thus affect the biological activity of the whole molecule. That activity is usually referred to the presence of electron-withdrawing substituents on the anilide moiety [14,20]. In accordance with these findings, our previous results revealed the same relation between the toxicity of ring-substituted hydroxynaphthanilides to the THP-1 cancer cells and the presence of substituents with electron-withdrawing properties [3,4,5]. The SAR studies also found the presence of an electron-withdrawing nitro group to be one of the essential requirements for the anticancer effect of niclosamide [21]. Based on these findings, the substitution by a nitro moiety was determined to be appropriate for the potent anticancer effect of newly-designed hydroxynaphthanilides.

Therefore, we have selected six newly-designed hydroxynaphthanilides, nitro-substituted in different positions on the anilide ring (Table 1), to evaluate their potential anticancer effects in the context of these structural differences. The aim of this work was to assess their antiproliferative activity in two cancer cell lines, THP-1 and MCF-7. Moreover, we also examined the effect on the growth of non-tumour cells 3T3-L1. In addition, changes in cell cycle distribution were evaluated, as well as their pro-apoptotic effect.

## 2. Results

### 2.1. Effect on Cell Proliferation and Viability

**Figure 1:**
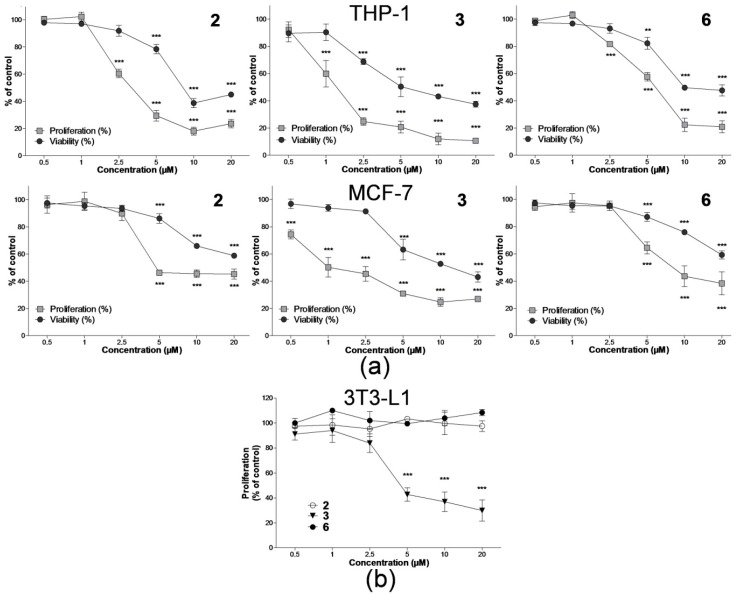
Effect of compounds **2**, **3**, and **6** on cell proliferation and viability in THP-1, MCF-7 and 3T3-L1 cell lines. Cells were cultured with indicated concentrations of compounds **2**, **3**, and **6** for 24 h. (**a**) Proliferation of THP-1 and MCF-7 cells was determined using WST-1 assay; cell viability was assessed by erythrosin B exclusion test; (**b**) Proliferation of 3T3-L1 cells was determined using WST-1 assay. The results are shown as the means ± standard deviation (SD) of three independent experiments, each performed in triplicate. ** *p* < 0.01, *** *p* < 0.001, statistically significant difference in comparison with drug-free control (CTRL).

Initially, we examined the effect of six nitro-substituted hydroxynaphthanilides on the proliferation of human leukaemia and breast carcinoma cell lines, using Water Soluble Tetrazolium Salts-1 (WST-1) assay. For such analyses, THP-1 and MCF-7 cells were treated with the compounds at concentrations ranging from 0.5 to 20 μM for 24 h. As shown in Figure 1a, compounds **2**, **3**, and **6** inhibit cell growth in both cell lines in a dose-dependent manner. The inhibitory effect of **2** and **6** was statistically significant (*p* < 0.001) starting from the concentration of 2.5 and 5 μM in THP-1 and MCF-7 cells, respectively. From the concentration-response curves, the IC_50_ values were determined. As summarized in Table 2, the IC_50_ values were found to be 3.06 μM in THP-1 and 4.61 μM in MCF-7 cells for compound **2**, and 5.80 and 5.23 μM in THP-1 and MCF-7 cells, respectively, for compound **6**. The strongest antiproliferative effect was observed in both THP-1 and MCF-7 cell lines after the treatment with compound **3** (IC_50_ 1.05 and 1.65 μM, respectively). In contrast, neither compound **1** nor **4** (both *ortho*-substituted derivatives) was able to induce the inhibition of cell growth in THP-1 or MCF-7 cells at concentrations used in the assay. Compound **5** demonstrated antiproliferative activity only in MCF-7 cells, significant (*p* < 0.001) at concentrations of 10 and 20 μM (data not shown), however, a 50% reduction in cell growth was not achieved. The proliferation of THP-1 cells was not affected by this compound.

After we found that compounds **2**, **3**, and **6** effectively inhibit the growth of both THP-1 and MCF-7 cancer cells at micromolar concentrations, we assessed additionally their effect on proliferation of non-tumour cell line, 3T3-L1, using WST-1 assay. While compounds **2** and **6** did not decrease cell growth at any of concentrations used, compound **3** affected the proliferation of 3T3-L1 cells in a dose-dependent manner (IC_50_ 4.41 μM) (Figure 1b and Table 2).

Subsequently, for the comparison of the antiproliferative and cytotoxic effects we assessed the cell viability after 24 h treatment with compounds **1**–**6** in both tumour cell lines using the dye exclusion test. In THP-1 cells, we obtained lower LC_50_ values: 7.91, 3.44, and 9.98 μM for compounds **2**, **3**, and **6**, respectively. In general, less sensitivity towards the cytotoxic effect of tested compounds was observed in MCF-7 cells. Neither compound **2** nor **6** reduced cell viability under 50% in comparison with the control, while the strongest effect was induced by compound **3** (LC_50_ 12.91 μM).

### 2.2. Effect on Distribution of Cells in Cell Cycle Phases

**Figure 2:**
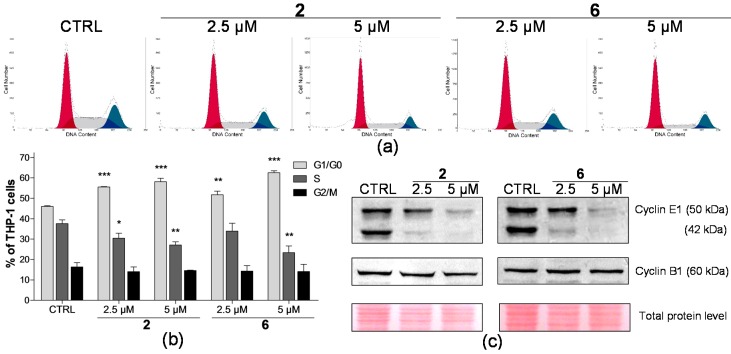
Compounds **2** and **6** induce accumulation of THP-1 cells in the G1 phase. (**a**) Representative histograms of flow cytometric analysis of the DNA content in THP-1 cells after the incubation with indicated concentrations of compounds **2** and **6** for 24 h; (**b**) The distribution of THP-1 cells in the phases of the cell cycle upon the treatment with compounds **2** and **6** at 24 h. The results are expressed as the means ± SD of three independent experiments. * *p* < 0.05, ** *p* < 0.01, *** *p* < 0.001, statistically significant difference in comparison with control sample; (**c**) Expression of cell cycle regulators cyclin E1 and B1 in THP-1 cells treated by compounds **2** and **6** for 24 h, as determined by Western blot analysis. Protein levels of the samples were normalized according to the total protein stains. CTRL, control cells treated by the drug-free medium.

**Figure 3:**
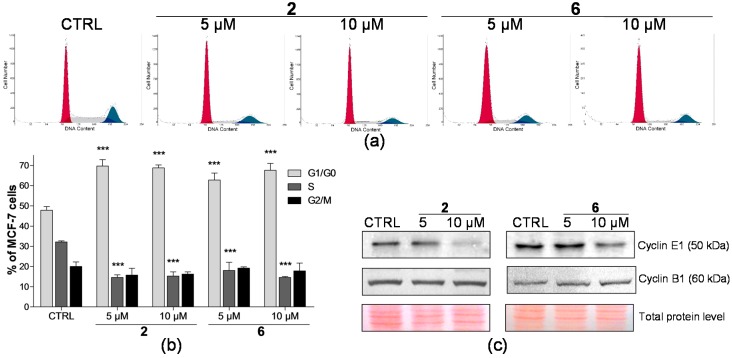
Compounds **2** and **6** induce accumulation of MCF-7 cells in the G1 phase. (**a**) Representative histograms of flow cytometric analysis of the DNA content in MCF-7 cells after the incubation with indicated concentrations of compounds **2** and **6** for 24 h; (**b**) The distribution of MCF-7 cells in phases of the cell cycle upon the treatment with compounds **2** and **6** at 24 h. The results are expressed as the means ± SD of three independent experiments. *** *p* < 0.001, statistically significant difference in comparison with control sample; (**c**) Expression of cell cycle regulators cyclin E1 and B1 in MCF-7 cells treated by compounds **2** and **6** for 24 h, as determined by Western blot analysis. Protein levels of the samples were normalized according to the total protein stains. CTRL, control cells treated by the drug-free medium.

The cell proliferation assays showed us the ability of selected compounds **2** and **6** to inhibit cancer cell growth. In order to determine at which stage of the cell cycle these compounds induce cell growth inhibition, flow cytometric analyses of cell cycle profiles in THP-1 and MCF-7 cell lines were performed. Cells were exposed to compounds **2** and **6** for 24 h at concentrations exerting significant inhibition of cell proliferation with no or very little concurrent effect on the cell viability. Therefore, THP-1 and MCF-7 cells were treated for 24 h with the compounds at concentrations of 2.5, 5, and 10 μM, respectively. In general, we detected a qualitatively similar effect on the distribution of cells in cell cycle phases following the treatment with compounds **2** and **6** in both leukaemia and breast carcinoma cells. Compounds **2** and **6** induced accumulation of cells in G1 phase in both THP-1 (Figure 2) and MCF-7 (Figure 3) cell lines. This was in concert with a simultaneous decrease in the number of cells observed in the S phase compared to the drug-free control, while the percentage of cells in the G2/M phase remained unchanged.

**Figure 4:**
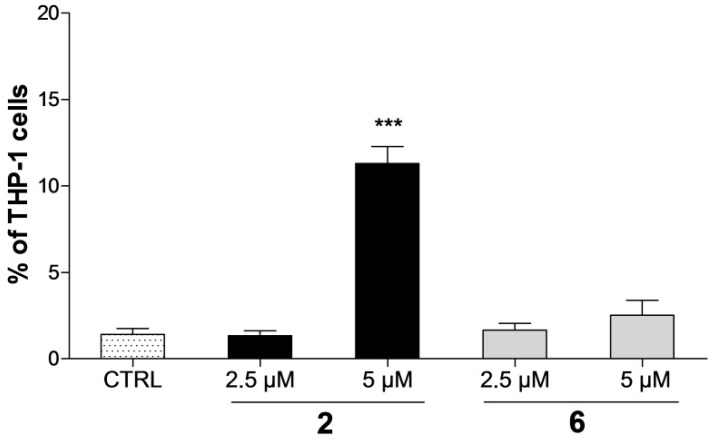
Compound **2** causes a significant increase of hypodiploid sub-G1 peak in THP-1 cells. Quantification of the sub-G1 peak in THP-1 cells after the treatment by compounds **2** and **6** for 24 h. The results are expressed as the means ± SD of three independent experiments. *** *p* < 0.001, statistically significant difference in comparison with the drug-free control (CTRL).

Additionally, the cell cycle analysis allows determining the presence of a subdiploid cell population as a characteristic marker of cells with fractional DNA content. A significant increase (*p* < 0.001) of the sub-G1 peak was found only after the treatment with 5 µM of compound **2** in THP-1 cells, where an approximately eight-fold increase was observed compared to the drug-free control (Figure 4). In contrast, compound **2** did not induce any elevation of the sub-G1 peak in breast carcinoma cells. Similarly, no significant increase of sub-diploid population of THP-1 or MCF-7 cells caused by 24 h treatment with compound **6** in comparison with the control sample was detected. Next, based on the flow cytometric data that showed the accumulation of cells in the G1 phase upon the treatment with compounds **2** and **6**, we examined their effect on the expression of regulatory proteins controlling G1/S and G2/M progression. Whereas total protein levels of cyclin B1 were not changed in THP-1 or MCF-7 cells, the treatment with both compounds **2** and **6** led to the dose-dependent decrease in expression of cyclin E1 (Figure 2c and Figure 3c). Importantly, the levels of cyclin E1 low molecular weight (LMW E1) isoform (42 kDa) were found to be significantly decreased in THP-1 cells.

### 2.3. Detection of Apoptosis by Annexin V-FITC/PI Assay

To further examine possible pro-apoptotic effect of compound **2** on THP-1 cells, Annexin V-FITC/PI assay was performed using flow cytometry for the quantification of the early and late stages of apoptosis. Staining of cells by Annexin V-FITC conjugate reflects the externalization of phosphatidylserine on the outer surface of the cell membrane as one of the early indicators of apoptosis [22]. In order to obtain further insight into the mechanism of cell death induced by compound **2**, we exposed THP-1 cells to a wider concentration range of 2.5, 5, and 10 µM and subsequently analysed the effect at three time-points of incubation (12, 18, and 24 h).

**Figure 5:**
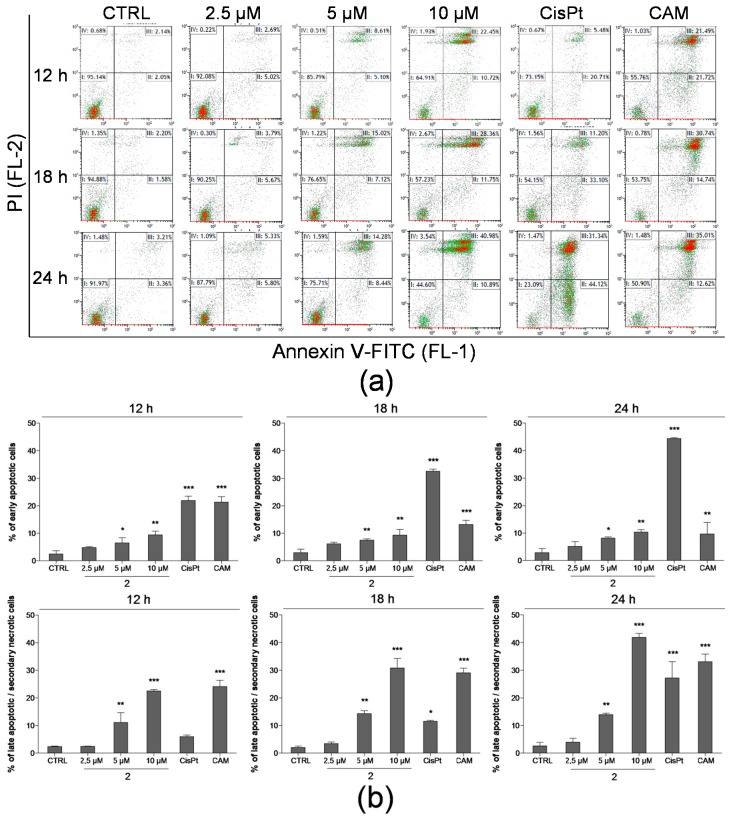
Detection of apoptosis after treatment with compound **2** in THP-1 cells at three points of incubation (12, 18, and 24 h). Cells were stained by Annexin V-FITC conjugate and PI; subsequent analysis was performed by flow cytometry. Cisplatin (10 µg/mL) and camptothecin (5 µM) were used as model compounds. (**a**) Representative dot plots of Annexin V-FITC/PI assay are shown. The particular quadrants represent proportion of cells that are I: viable; II: early apoptotic; III: late apoptotic/secondary necrotic; IV: necrotic; (**b**) Proportion of early apoptotic and late apoptotic/secondary necrotic THP-1 cells after the treatment by compound **2** and model compounds. The results are expressed as the means ± SD of three independent experiments. * *p* < 0.05, ** *p* < 0.01, *** *p* < 0.001, statistically significant difference in comparison with the drug-free control (CTRL).

The assay revealed that compound **2** induced a dose-dependent increase of the percentage of early apoptotic as well as late apoptotic/secondary necrotic leukaemia THP-1 cells. In correspondence with the previous detection of a subdiploid cell population compound **2**, at concentrations of 2.5 µM, 5 µM, and 10 µM, elicited elevations of Annexin V/FITC-stained cell populations. As shown in Figure 5, this effect was observed even after 12 h of incubation; 10 µM of compound **2** increased significantly (*p* < 0.01) the proportion of early apoptotic cells to 9.37% in comparison to the percentage of control cells, 2.41%. The same concentration of compound **2** induced the elevation of the number of double-stained cells with incubation time, from 22.48% after 12 h to 41.88% after 24 h incubation. In general, the percentage of late apoptotic/secondary necrotic cells at higher concentrations of compound **2** (5 and 10 µM) prevailed over the early apoptotic cell population at all determined time points.

Nevertheless, the different effect was observed after the treatment with two model compounds exerting the pro-apoptotic effect in THP-1 cells. As summarized in Figure 5, cisplatin was found to most effectively increase the rate of early apoptotic cells in a time-dependent manner up to 44.38% after 24 h exposure. While camptothecin increased significantly (*p* < 0.001) the percentage of both early and late apoptotic cells up to 21.28% and 24.10%, respectively, after 12 h, 24 h treatment led to a decrease of the early apoptotic population to 9.65%; in contrast, late apoptosis increased to 33.08%.

### 2.4. Analysis of Proteins Levels Involved in Apoptotic Pathways

**Figure 6:**
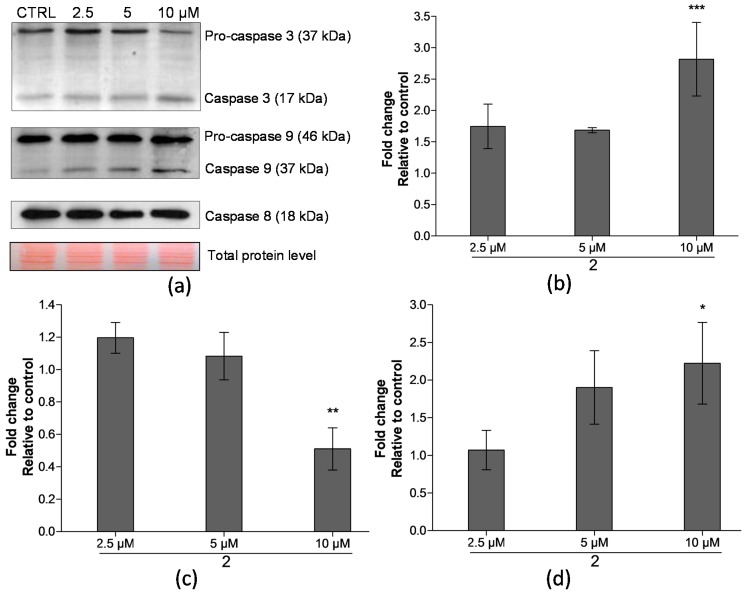
Levels of proteins involved in apoptotic pathways in THP-1 cells after 24 h treatment by compound **2**. (**a**) Levels of caspase 3, caspase 8, and caspase 9 in THP-1 cells treated by compound **2** for 24 h, as determined by Western blot analysis. Data of typical immunoblot are reported; (**b**) Summary data of cleaved caspase 9 levels in THP-1 cells; (**c**) Summary data of pro-caspase 3 levels in THP-1 cells; (**d**) Summary data of cleaved caspase 3 levels in THP-1 cells. Protein levels of the samples were normalized according to the total protein stains. The results are expressed as the means ± SD of three independent experiments. * *p* < 0.05, ** *p* < 0.01, *** *p* < 0.001, statistically significant difference in comparison with the drug-free control (CTRL).

Most of the apoptotic signalling pathways are controlled by caspases that belong to a group of cysteine proteases [23]. To assess whether compound **2** affects these signalling cascades and which pathway is activated (intrinsic or extrinsic), the activities of caspase 3, caspase 9, and caspase 8 were evaluated using Western blot analysis. As summarized in Figure 6, after 24 h incubation, compound **2** induced cleavage of pro-caspase 3 dose-dependently; an approximately two-fold decrease of the inactive form upon the treatment with 10 μM compared to the control was detected. Similarly, a comparable two-fold increase of active caspase 3 level was observed after the exposure to the 10 μM concentration of compound **2** in comparison to the control. Additionally, a significant increase of cleaved caspase 9 levels was detected with the most pronounced effects at 10 μM. On the contrary, the level of active caspase 8 was not altered after the treatment with compound **2** in comparison to the control.

## 3. Discussion

In the present study, we examined the anticancer effects of a series of newly-synthesized nitro-substituted hydroxynaphthanilide derivatives through the assessment of their antiproliferative activity and cytotoxicity.

Our results showed the difference among the tested compounds in the antiproliferative activity. We found that the potency of cell growth inhibition correlates with the position of the electron-withdrawing nitro group on the anilide ring of the tested compounds. While *ortho*-substituted derivatives did not elicit any antiproliferative effect in both THP-1 and MCF-7 cancer cells, the shift of the nitro group to the *meta*- or *para*-position in compounds **2**, **3**, and **6**, led to the cell growth inhibition. Thus, it can be assumed that, most likely, the antiproliferative activity of 3-hydroxynaphthalene-2-carboxanilide and 2-hydroxynaphthalene-1-carboxanilide derivatives increase depending on the position of the nitro group as follows: *ortho* < *meta* < *para*. This different activity could be possibly related to the steric effect of the anilide substituents. Recently, it was described that the presence of a substituent in the *ortho* position causes the twist of the whole aniline ring plane towards the naphthalene scaffold, while *meta*- and, especially, *para*-substituted derivatives have a practically linear molecule [24]. Moreover, not only the location of the substituent on the anilide moiety but also the position of the β-ring of naphthalene towards the phenolic and carboxanilide moietis affected the intensity of the antiproliferative effect of these compounds. In our study, stronger antiproliferative activity was observed in substituted 3-hydroxynaphthalene-2-carboxanilides when comparing the IC_50_ values of *meta*-substituted compounds **2** and **4** or *para*-substituted **3** and **6**. The similar structure-activity relationship was determined for the cytotoxicity of the tested compounds. Nevertheless, compounds **2**, **3**, and **6** exerted stronger antiproliferative rather than cytotoxic effect in cancer cells; approximately 2–3-fold higher LC_50_ values compared to IC_50_ values were obtained in the assays on THP-1 cells. Even more pronounced difference was observed in MCF-7 cells, where the LC_50_ values were achieved only upon the treatment with compound **3**, with an approximately seven-fold higher dose in comparison with IC_50_.

To assess whether tested compounds also influence the growth of other than cancer cells, we have extended our antiproliferative analysis and employed non-tumour fibroblast cell line 3T3-L1. Compound **3** that exerted the most substantial antiproliferative and cytotoxic effects towards both cancer cell lines was also capable of inhibiting the growth of the non-tumour line. Interestingly, a different effect was observed upon the treatment with compounds **2** and **6**, where such antiproliferative activity in non-tumour cells was not detected. Results of antiproliferative effects showed us that among all tested compounds, compounds **2** and **6** were the most potent and, thus, were chosen for further, more detailed analyses.

One characteristic feature of cancer cells is the deregulation of the cell cycle, which leads to their uncontrolled proliferation. Therefore, the inhibition of cell cycle progression represents a common target of anticancer agents [25]. We performed the cell cycle analysis to reveal whether the antiproliferative effect of compounds **2** and **6** is reflected in the modification of cell cycle progression. Our results showed that both compounds were able to accumulate THP-1 and MCF-7 cancer cells in the G1 phase and to inhibit the transition of cells to the synthetic phase. We assume that this most likely reflects the antiproliferative effect observed in both cell lines (Figure 1a). The progression through the cell cycle is mediated by a family of cyclin-dependent kinases, the activity of which depends on the binding of the regulatory proteins, cyclins [26]. The observed accumulation of THP-1 and MCF-7 cells in the G1 phase after the treatment with compounds **2** and **6** was accompanied by a reduction of cyclin E1 level in a dose-dependent manner (Figure 2c). As the activator of CDK2, cyclin E1 is responsible for the G1/S phase progression and, thus, it is involved in surpassing the restriction point [27]. Many cancers typically overexpress cyclin E1, which is also proved in the MCF-7 cell line [28]. This might support our finding of only slight downregulation of cyclin E1 caused by the treatment of MCF-7 cells with compounds **2** and **6**, although these compounds effectively inhibited the G1/S transition. Interestingly, besides the downregulation of cyclin E1 full-length form, we also detected a more pronounced reduction of LMW E1 isoform levels in THP-1 cells treated with compounds **2** and **6**. LMW E1 isoforms are generated primarily in cancer cells, where they still remain fully functional. They have even higher potency to increase CDK2/E1 activity than the full-length form and, thus, they move the cells through the cell cycle more effectively than the full-length form [29,30]. Our previous study reported a similar detection of the decreased levels of cyclin E1 isoforms in THP-1 cells treated with geranylated flavanone tomentodiplacone B that coincided with an induced accumulation of cells in G1 phase [31]. While cyclin B1 is involved in the G2/M transition associated with CDK1 [26], we did not observe any change in the levels of cyclin B1 in THP-1 or MCF-7 cells after the exposure to compounds **2** and **6**. These findings are supported by our flow cytometric data that did not indicate any significant difference in the proportion of cells in G2/M cell cycle phase upon the treatment with these compounds (Figure 2b and Figure 3b). Based on those results, we could suggest that compounds **2** and **6** most likely affect G1/S rather than the G2/M transition.

The presence of cell nuclei with hypodiploid DNA content during the cell cycle analysis could indicate a possible presence of apoptotic cells [32]. The assessment of sub-G1 peak levels revealed different effects among the tested compounds; a significant increase was detected only in THP-1 cells upon the treatment with compound **2** (Figure 4). Based on these findings, we performed further analysis to prove its possible pro-apoptotic effect in the THP-1 cell line. Results of Annexin V-FITC/PI assay showed us that compound **2** induced the THP-1 cells to undergo an early stage of apoptosis even after 12 h exposure (Figure 5). Nevertheless, compound **2** accumulated more effectively (dose and time-dependently) in cells in the late apoptotic stage. These results correlate with the data obtained from the viability staining assay. In addition, two already known anticancer agents of a different mode of action, cisplatin, which is able to crosslink with the DNA and, thus, cause DNA damage [33], and camptothecin as the S-phase-specific inhibitor of the enzyme DNA topoisomerase-I [34], were added to the assay as model compounds with proved pro-apoptotic effects in THP-1 cells [35,36]. Although our results found all three compounds to significantly increase the number of cells positive for Annexin V-FITC staining, their effect led to different proportions of early and late apoptotic/secondary necrotic cells. While cisplatin induced a time-dependent substantial increase in the fraction of early apoptotic cells, camptothecin most likely elicited the time-dependent transfer of cells from early apoptotic to late apoptotic stages. These differences observed in the effect of three tested compounds enable us to presume a different mechanism of action of compound **2** in comparison with one of the two model anticancer agents.

These findings prompted us to further investigate the involvement of compound **2** in the apoptotic pathways. The caspases regulate the process of apoptosis in a different manner. The activation of caspase 8 is realized through the extrinsic apoptotic pathway after the binding of a ligand to an appropriate death receptor. Subsequently, the active form interacts with effector caspase 3 and that results in its cleavage and activation. On the other hand, initiator caspase 9 is involved in the intrinsic, also known as the mitochondrial apoptosis pathway, and is activated after the leakage of the mitochondrial cytochrome c. This also leads to proteolytic cleavage of inactive procaspase 3 and to its activation. Therefore, it denotes the essential role of caspase 3 in both extrinsic and intrinsic pathways, as it also comprises a link between them [37,38]. After 24 h treatment, compound **2** was found to be capable of inducing an increase of active caspase 3 level, including the decreased level of inactive pro-caspase 3, both significantly at a concentration of 10 µM (Figure 6). At the same time, compound **2** caused also the cleavage of pro-caspase 9. On the contrary, no change in the level of the active form of caspase 8 was observed in comparison with the control, non-treated cells. These results indicate that compound **2** induces apoptosis in THP-1 cells by activating a caspase cascade. In addition, we could hypothesize that this compound might be preferably involved in the intrinsic apoptotic pathway. However, such specificity needs to be proved by additional analyses, and the mechanism of targeting apoptotic pathway remains unknown.

## 4. Materials and Methods

### 4.1. Chemicals and Reagents

The tested nitro-substituted hydroxynaphthanilides **1**−**6** were prepared and supplied by the Department of Chemical Drugs, Faculty of Pharmacy, University of Veterinary and Pharmaceutical Sciences Brno, Czech Republic. The synthesis and structural characterization of these compounds have been described previously [3,5]. Due to poor solubility in water, the compounds were dissolved in dimethyl sulfoxide (DMSO) (Sigma-Aldrich, St. Louis, MO, USA), while the stock solutions were prepared freshly before each experiment. The final concentration of DMSO in the assays never exceeded 0.1% (*v*/*v*). Cisplatin and camptothecin were purchased from Sigma-Aldrich. RPMI 1640 and DMEM culture media, phosphate-buffered saline (PBS), foetal bovine serum (FBS) and antibiotics (penicillin and streptomycin) were obtained from HyClone Laboratories, Inc. (GE Healthcare, Logan, UT, USA). Mouse monoclonal antibodies against cyclin E1 (sc-247), caspase 3 (sc-7272) and caspase 9 (sc-17784) were purchased from Santa Cruz Biotechnology (Santa Cruz, CA, USA). Rabbit polyclonal antibodies against cyclin B1 (ab2949) and caspase 8 (ab-25901) were purchased from Abcam (Cambridge, UK). All other reagents, unless specified elsewhere, were purchased from Sigma-Aldrich.

### 4.2. Cell Culture

THP-1 human monocytic leukemia cell line, MCF-7 human breast adenocarcinoma cells and 3T3-L1 mouse embryonic fibroblast were purchased from the European Collection of Cell Cultures (ECACC, Salisbury, UK). Cells were routinely tested for the absence of mycoplasma (Hoechst 33258 staining method). THP-1 cells were maintained in RPMI 1640 culture medium containing 2 mM l-glutamine; MCF-7 and 3T3-L1 cells were cultured in DMEM medium. All of the culture media were supplemented with 10% heat-inactivated FBS and antibiotics (100 U/mL penicillin and 100 μg/mL streptomycin). Cells were maintained at 37 °C in a humidified atmosphere containing 5% CO_2_.

### 4.3. Analysis of Cell Proliferation and Viability

Cell proliferation was evaluated using Cell Proliferation Reagent WST-1 (2-(4-iodophenyl)-3-(4-nitrophenyl)-5-(2,4-disulfophenyl)-2*H*-tetrazolium) (Roche Diagnostics, Mannheim, Germany) according to the manufacturer´s instructions. THP-1 (5 × 10^4^ cells/100 μL culture medium per well), MCF-7 cells (1 × 10^4^ cells/100 μL per well), and 3T3-L1 (2.5 × 10^3^ cells/100 μL per well) were cultured in 96-well plates in triplicate. The measurement was performed using Synergy 2 Multi-Mode Microplate Reader (BioTek, Winooski, VT, USA) after 24 h incubation of cells with tested compounds dissolved in DMSO and subsequently in RPMI 1640 to final concentration ranging 0.5–20 μM in the assays. Cell viability was assessed by dye exclusion test. THP-1 (2 × 10^5^ cells/mL per well) and MCF-7 cells (8 × 10^4^ cells/mL per well) were incubated in 24-well plates with the indicated concentrations of compounds for 24 h. The number of viable cells was determined using hemocytometer after their staining with a solution of erythrosin B (0.1% erythrosin B (*w/v*) in PBS). The assays were conducted in triplicate. The IC_50_ and LC_50_ values were calculated from fitted concentration-response curves using GraphPad Prism 5.00 software (GraphPad Software, San Diego, CA, USA).

### 4.4. Cell Cycle Analysis

THP-1 and sub-confluent MCF-7 cells were treated and subsequently incubated with indicated concentrations of compounds **2** and **6** for 24 h. After the incubation, cells were washed twice in PBS (pH 7.4), fixed in 70% ethanol and stored at −20 °C overnight. Fixed cells were collected by centrifugation, and supernatant was discarded. The cell pellet was washed twice with PBS and incubated with RNaseA (0.02 mg/mL) and 0.05% (*v*/*v*) Triton X-100 in PBS for 30 min at 37 °C. After the nuclei staining with propidium iodide (PI) (0.04 mg/mL), the cell cycle distribution was analysed using a flow cytometer Cell Lab Quanta SC (Beckman Coulter, Brea, CA, USA). The quantification of cell cycle distribution was carried out using software MultiCycle AV (Phoenix Flow System, San Diego, CA, USA). A total number of 2 × 10^4^ cells was analysed per sample.

### 4.5. Detection of Apoptosis Using Annexin V-FITC/PI Assay

Early and late stages of apoptosis were detected using Annexin V-FITC Kit—Apoptosis Detection Kit faccording to the manufacturer´s instructions. THP-1 cells were treated with increasing concentrations of compound **2** (2.5, 5, and 10 μM), cisplatin (10 μg/mL) and camptothecin (5 μM). At each time-point of incubation (12, 18, and 24 h) the cells were washed with ice-cold PBS prior to being resuspended at a concentration of 5 × 10^6^ cells/mL in a total volume of 100 μL of 1× binding buffer. Annexin V-FITC solution (final concentration 0.25 μg/mL) and PI (final concentration 12.5 μg/mL) were added to each sample; the cell suspension was kept on ice and incubated for 15 min in the dark. After that, the analysis was carried out by flow cytometry. The data were evaluated using Kaluza Flow Cytometry Analysis 1.2. Per sample, a total number of 2 × 10^4^ cells were analysed.

### 4.6. Western Blotting

For Western blotting, cells were washed with PBS and lysed in lysis buffer (100 mM Tris-HCl, pH = 6.8; 20% glycerol; 1% SDS) containing protease and phosphatase inhibitor cocktails. Protein concentration was measured using Roti^®^-Quant universal (Carl Roth, Karsruhe, Germany) according to the manufacturer’s instructions. Cell lysates were supplemented with bromophenol blue (final concentration 0.01% (*w*/*v*)) and β-mercaptoethanol (final concentration 1% (*v*/*v*)) prior to being heated for 5 min at 95 °C. Equal amounts of protein (10 μg) were loaded into a 12% polyacrylamide gel, separated by SDS-polyacrylamide gel electrophoresis and subsequently electrotransferred onto nitrocellulose membranes. Reversible Ponceau S. staining was performed to assess equal sample loading. Then, the membranes were blocked with 5% non-fat dry milk in TBST (10 mM Tris-HCl pH = 7.5, 150 mM NaCl, 0.1% (*v/v*) Tween-20) and appropriate primary and secondary antibodies were used for immunodetection. The proteins were visualized by ECL Plus reagent according to the manufacturer’s instructions. The intensity of bands was semi-quantitatively analysed using the ImageJ software (National Institute of Mental Health, Bethesda, MD, USA).

### 4.7. Statistical Analysis

All experimental data were expressed as the arithmetical mean ± standard deviation (SD). Statistical analysis was performed using one-way analysis of variance (ANOVA) followed by the Dunnett’s post test using GraphPad Prism 5.00 software. Statistical significance was assessed at levels of *p* < 0.05, *p* < 0.01 and *p* < 0.001.

## 5. Conclusions

The present study provides the first description of the antiproliferative activity of nitro-substituted hydroxynaphthanilides in the context of structure-activity relationships. Our results indicate that the potency of ring-substituted hydroxynaphthanilides towards cell growth inhibition increases with positioning of the nitro group as follows: *ortho* < *meta* < *para*. The most promising compounds **2** and **6** exerted antiproliferative activity in THP-1 and MCF-7 cancer cells with single-digit micromolar IC_50_ values, while they had a minimal effect on the growth of 3T3-L1 non-tumour cells. Compounds **2** and **6** accumulated cancer cells THP-1 and MCF-7 in G1 cell cycle phase, which was accompanied by the observed down-regulation of cyclin E1 levels. Moreover, compound **2** was found to induce apoptosis in THP-1 cells via a caspase-mediated cascade. The results also indicate that apoptosis was probably induced through the intrinsic apoptotic pathway, although further analysis is still required to verify such assumption. According to the results, nitro-substituted hydroxynaphthanilides **2** and **6** can be considered as potential anticancer agents, and the structure of hydroxynaphthanilides is an appropriate model moiety for further design of compounds with potential anticancer properties.

## Figures and Tables

**Table 1:** Structures of tested compounds: (**a**) 3-hydroxy-*N*-(nitrophenyl)naphthalene-2-carboxamides; and (**b**) 2-hydroxy-*N*-(nitrophenyl)naphthalene-1-carboxamide.

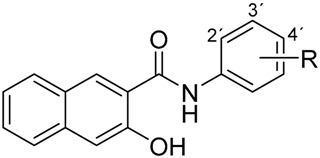		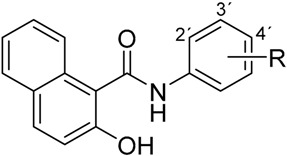	
(a)		(b)	
Compound	R	Compound	R
**1**	2-NO_2_	**4**	2-NO_2_
**2**	3-NO_2_	**5**	3-NO_2_
**3**	4-NO_2_	**6**	4-NO_2_

**Table 2:** Antiproliferative and cytotoxic effects of tested compounds **1**−**6**. IC_50_ and LC_50_ values were calculated using concentration-response curves generated from the results of WST-1 analysis and erythrosin B exclusion test, respectively. The values represent means ± SD of three independent experiments, each performed in triplicate.

Compound	THP-1		MCF-7		3T3-L1
	IC_50_ (μM)	LC_50_ (μM)	IC_50_ (μM)	LC_50_ (μM)	IC_50_ (μM)
**1**	>20	>20	>20	>20	>20
**2**	3.06 ± 0.206	7.91 ± 0.240	4.61 ± 0.068	>20	>20
**3**	1.05 ± 0.199	3.44 ± 1.209	1.65 ± 0.938	12.91 ± 1.984	4.41 ± 0.293
**4**	>20	>20	>20	>20	>20
**5**	>20	>20	>20	>20	>20
**6**	5.80 ± 0.370	9.98 ± 0.349	5.23 ± 0.802	>20	>20
